# Gut Microbiota: An Important Link between Western Diet and Chronic Diseases

**DOI:** 10.3390/nu11102287

**Published:** 2019-09-24

**Authors:** Zumin Shi

**Affiliations:** Human Nutrition Department, College of Health Science, QU Health, Qatar University, Doha 2713, Qatar; zumin@qu.edu.qa; Tel.: +974-4403-6037

A Western diet characterised by high intake of energy-dense and processed food is a risk factor for many chronic diseases including diabetes, obesity and cardiovascular diseases [1]. Much of the research focus is on the high intake of fat, sugar, and low intake of fibre and fruits and vegetables [1]. One of the underlying mechanisms linking Western diet and chronic diseases is inflammation. A dietary inflammatory index has been proposed and tested to reflect the link between diet and inflammation in different populations [2]. 

Our diet provides not only the nutrients we need but also the material and medium for sustenance and growth of gut bacteria. The composition of the diet will inevitably affect selective growth of types of bacteria in the gut. Since the role of gut microbiota in health has been discovered [3], there is a growing number of studies on the role of diet on gut microbiota [4,5,6,7]. Among the studied foods, ultra-processed food has attracted great attention. The concept of ultra-processed foods came from the NOVA system. According to the NOVA classification system, foods are classified into four groups based on the extent and purpose of the industrial processing involved [8]. Ultra-processed foods (e.g., soft drinks, savoury snacks) are highly palatable, having long shelf-life and relatively cheap and can be consumed anywhere at any time [8]. However, these foods are typically characterized by a poor nutritional profile. The consumption of ultra-processed food is high in many high income countries. For example, the percentage of energy intake from ultra-processed food is 29.1% in France [9], 42% in Australia [10] and 57.9% in the USA [11]. 

Zinöcker MK and Lindseth IA systematically examined the effects of ultra-processed food on gut microbiota [12]. The review is ground-breaking in several respects. It is the first comprehensive review of the current evidence on the impact of ultra-processed food on gut microbiota, mainly conducted in animals. A list of harmful factors of ultra-processed food has been reviewed and suggests the significance and magnitude of the problems. The factors causing concern include acellular nutrients, food additives (e.g., emulsifiers and artificial sweeteners) and pathogen associated molecular patterns (PAMPs). The effects of ultra-processed foods on human health are likely to be the result of synergic effects of many compounds and characteristics of the foods. The key message from the review is that high consumption of ultra-processed food can change the gut microbiota and lead to inflammation. The effects can even be transferred to later generations via epigenetic change. As the review is mainly based on animal studies, more research is needed in humans, especially long-term clinical trials [13]. 

Although Zinöcker MK and Lindseth IA have provided a comprehensive list of risk factors that may affect the gut microbiota [12], the list may keep increasing with the advance of our understanding of ultra- processed food and the use of new food technology. This review should serve as a wakeup call for the action on regulating ultra-processed food. Before approval, there is no regulation for the testing of the effects of food additives on gut microbiota. Although some food additives can be beneficial for human health, others may alter the composition of the microbiota and lead to gut inflammation, which may promote diverse forms of inflammatory diseases. 

Data from population based epidemiological studies consistently suggest a strong association between ultra-processed food and health outcomes in different countries. In 2019 alone, four large-scale studies have been published. Data from the NutriNet-Santé cohort (France 2009–2018) study suggest that the association between ultra-processed food and CVD is independent of BMI, intake of energy, fat and fibre [14]. In the same cohort study, consumption of ultra-processed food was associated with an increased risk of mortality [9] and depression [15]. In Spain, findings from the SUN prospective cohort study suggest that for each additional serving of ultra-processed foods, all-cause mortality increased by 18% [16]. Although these studies did not test the mediating effect of gut microbiota based on the existing evidence in the field, the findings are likely to be true. The consumption of ultra-processed food has also been linked to urinary levels of phthalates and bisphenols in the U.S. National Health and Nutrition Examination Survey [17]. 

The evidence requires a change in our dietary guidelines by including guidelines on food processing. Current dietary guidelines in most of the countries focus on nutrients or food groups. However, still the Mediterranean diet is recommended for better health outcomes without taking into account to what extent the major food groups in the Mediterranean diet are processed [18]. Even home processing of these food may corrupt the health effects. For instance, frying vegetables with olive oil at high temperature is less likely to have anticipated health benefits. A vegetable-rich dietary pattern in which vegetables were cooked with oil was found to be associated with the risk of obesity in the Chinese population [19]. 

Making healthy foods from raw material available and affordable is essential to reduce the consumption of ultra-processed food. To achieve this goal, the government and the food industry should work together. Monetary policy should be in place to foster a healthy food environment. This includes putting a tax on ultra-processed food. Using gut microbiota as indicators for the assessment of food safety could be an effective way to regulate the production and amount of ultra-processed food in the market. However, more research is still needed before gut microbiota can be used in the assessment of food safety.
